# Exploring the perspective of young adults about anaemia prevention; the contributions of knowledge about at-risk groups and consequences of anaemia

**DOI:** 10.1186/s12889-023-16980-2

**Published:** 2023-10-24

**Authors:** Audrey Benfo, Francis Zumesew, Ebenezer Bugyei Akoto, Edward Ahiakwah, Belinda Baidoo, Nana Ama Frimpomaa Agyapong, Joseph Boachie, Patrick Adu

**Affiliations:** 1 Department of Medical Laboratory Sciences, School of Allied Health Sciences, University of Cape Coast, Cape Coast, Ghana; 2 Department of Clinical Nutrition and Dietetics, School of Allied Health Sciences, University of Cape Coast, Cape Coast, Ghana

**Keywords:** Anaemia, Consequences of anaemia, Causes of anaemia, Prevention of anaemia, Young adults

## Abstract

**Background:**

Anaemia persistently remains a grave public health challenge in most sub-Saharan African countries. Understanding the perspectives of young adults concerning the multi-factorial nature of anaemia may be an important step towards meeting the 2025 global nutrition target of halving anaemia since these individuals might be in the process of reproductive decisions.

**Aim:**

To explore the relationship between students’ knowledge about individuals at risk of developing anaemia, and anaemia consequences, and anaemia prevention strategies in a tertiary student cohort.

**Methods:**

This sequential exploratory study adopted a mixed-methods approach to triangulate the data collection. A semi-structured questionnaire was used to gather baseline data regarding students’ perspective on anaemia. Themes that emerged from the initial questionnaire data analyses guided a focus group discussion (FGD) to further explore students’ perspectives on anaemia. FGD data was thematically analysed to unearth reasons behind questionnaire item selection. Structural equation modeling (SEM) was used to explore the relationship between constructs in the anaemia knowledge questionnaire.

**Results:**

Overall, 543 students participated in the initial questionnaire data acquisition compared to 16 in the FGD. Our latent variable structural model showed that knowing the causes of anaemia did not significantly (*p* > 0.05) associate with either knowledge about anaemia consequences (b = 0.113) or knowledge about anaemia prevention strategies (b = 0.042). However, knowledge about individuals at-risk of anaemia was significantly positively associated with both anaemia prevention strategies (b = 0.306, *p* < 0.05) and knowledge about consequences of anaemia (b = 0.543, 95%). Moreover, knowing the consequences of anaemia seemed to significantly positively mediate the association between knowledge about at-risk groups and preventive measures that could be adopted (b = 0.410, *p* < 0.05).

**Conclusions:**

Systems thinking public health educational campaigns that highlight the consequences of anaemia and at-risk groups are more likely to inspire the adoption of preventive strategies among young adults.

**Supplementary Information:**

The online version contains supplementary material available at 10.1186/s12889-023-16980-2.

## Background

Globally, anaemia is estimated to affect more than three persons in every ten individuals, with developing countries accounting for > 80% of the global anaemia burden [1, 2]. In most sub-Saharan African countries, anaemia is a moderate or severe public health problem [3]. In Ghana, the prevalence of anaemia has been estimated among different sections of the population; 42% among women of reproductive age [4, 5], 13%—50% among adolescents [6, 7], 49%—66% among children under 5 years [5, 8], and 39% among the elderly [8, 9]. In a previous study that involved a cohort of university students, anaemia prevalence was estimated to have increased from 20% to 45.1% over the course of one academic year [10]. That study therefore found anaemia to be a moderate public health problem, at the beginning of the academic year, but anaemia prevalence progressed to a severe public health challenge among students during the latter parts of the second semester. Most of the previous anaemia-related studies in literature have focused on assessing anaemia and risk factors of anaemia [5, 11, 12]. However, there is scanty primary research exploring the perceptions of individuals on anaemia and what their knowledge of predisposition factors to the condition are [13]. This is in spite of the fact that anaemia is endemic in Ghana, and that misconceptions and/or poor knowledge about anaemia may be a hindrance to the successful implementation of any anaemia control strategies [14].

The aim of this study, therefore, was to explore the perceptions of young adults about anaemia and how perceptions and knowledge are interlinked. We sequentially employed both quantitative and qualitative data collection approaches to explore the perceptions of young adults about anaemia as a means of triangulating the data to increase scientific rigour as well as the generalizability of the study findings. Whereas the qualitative data collection enabled us to explore the perceptions of these young adults about anaemia, the quantitative data was used to establish relationships between the knowledge about causes of anaemia, consequences of anemia, and individuals at-risk of anaemia as well as the preventive measures students adopt to curb anaemia.

## Materials and methods

### Study design/setting

**Fig. 1:**
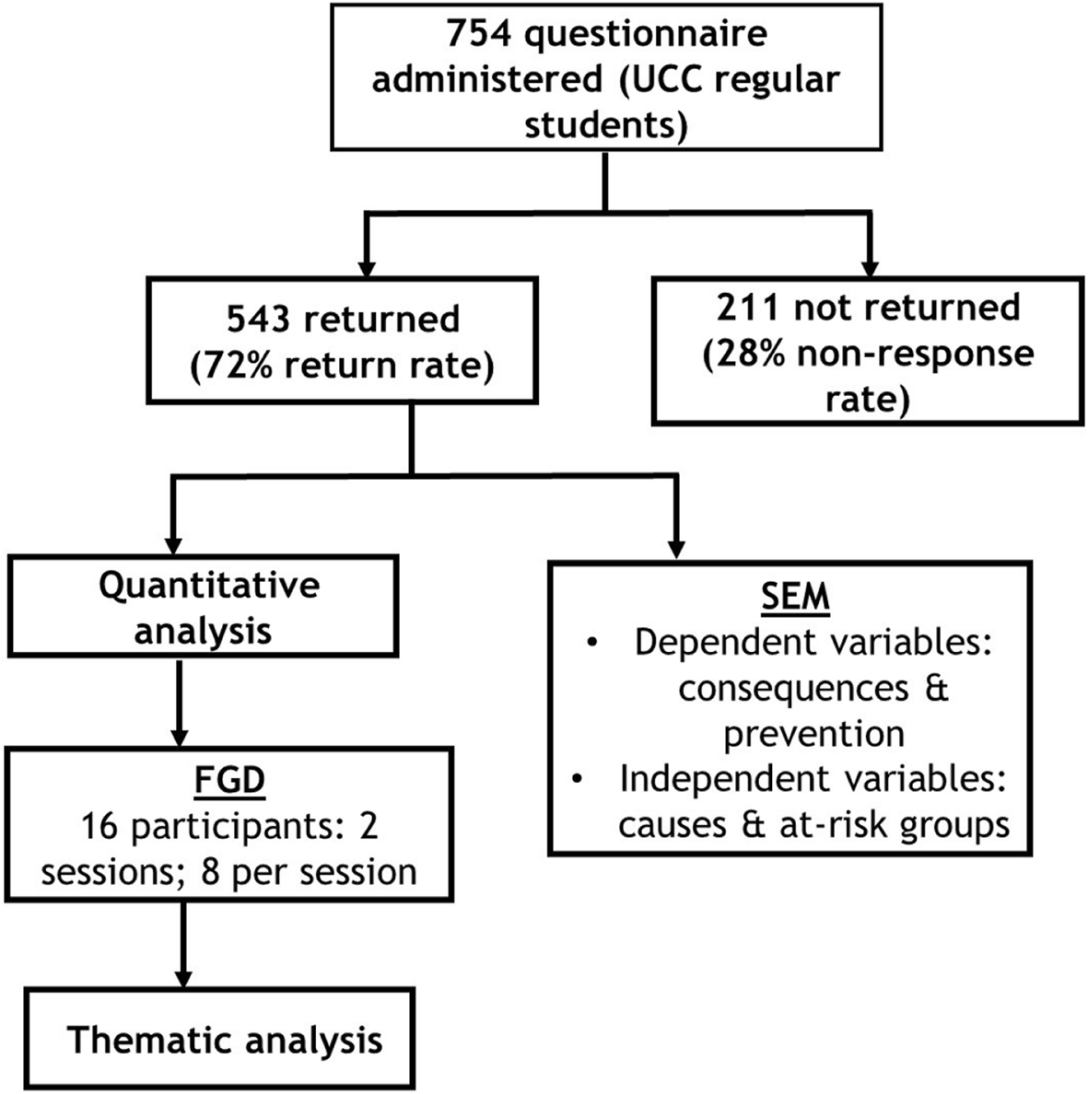
PRISMA diagram illustrating participant recruitment and data collection strategies. UCC: University of Cape Coast; SEM: structural equation modeling; FGD: focus group discussion

This was a sequential exploratory study that employed a mixed-methods approach to triangulate data collection. Specifically, the study sequentially used a semi-structured questionnaire to obtain baseline data from a sample of the University of Cape Coast students about their understanding of anaemia, and then followed up with a focus group discussion (FGD) of participants. Since the findings from the quantitative analyses of the semi-structured questionnaires informed the FGD, only individuals who had answered the questionnaires were invited to be part of the FGD. The study was undertaken from January – November 2022. Figure 1 is an illustration of the participant recruitment and data collection strategies employed.

### Sampling

A convenience sampling technique was employed to recruit study participants.

### Sample size calculation

At the time of the study, the total population of students of the University of Cape Coast (UCC) in the regular mode was 19,963; 18949 regular undergraduate students and 1014 regular postgraduate students. The required sample size was estimated to be 377 using a 95% confidence level, a population proportion of 50%, and a 5% margin of error. However, in a previous research that administered questionnaires to a cohort of UCC students [10], the authors recorded approximately a 50% return rate. Therefore, twice the estimated sample size (754 questionnaires) was initially distributed for the quantitative data collection. Overall, 543 questionnaires were returned indicating an improved return rate of 72% (compared to the 50% response rate in our previous publication) [10].

### Inclusion/exclusion criteria

The study targeted all regular undergraduate and postgraduate students of the University of Cape Coast who were willing to participate. However, students in the distance learning mode as well as all students on exchange programs from universities abroad were excluded from the survey because of ease of access and in view of the invitation for the follow up face-to-face focus group discussion.

### Questionnaire

A semi-structured questionnaire with two (2) sub-sections was used to collect data (Supplementary File SF1). Section 1 solicited background data, a definition of anaemia as well as an option to opt-in for the focus group discussion. Section 2 solicited information from participants concerning their respective dietary habits, knowledge about the causes of anaemia, individuals at-risk of developing anaemia, consequences of anaemia, and prevention strategies to curb anaemia. In the second section of the questionnaire, students judged their agreement with specific statements on a five-point Likert scale ranging from 1 (strongly disagree) to 5 (strongly agree). The questionnaire was initially piloted using a sample of 20 random students (approximately 5% of the required sample size) to validate the constructs prior to adoption for the large-scale data collection.

### Focus group discussion (FGD)

The FGD was the second phase of the data collection (the FGD guide was an adaptation from previous publication [13] and is enclosed as Supplementary File SF2). The inclusion criterion for the FDG was that only consented individuals who had previously answered the questionnaire were invited to be part of the FGD. Since the themes that informed the FGD emerged from the analyses of the questionnaire responses, only individuals who had answered the questionnaires could fully understand and participate meaningfully in the FGD. Each FGD took about 60 min and was conducted in English language since all the participants were tertiary students; however, participants were encouraged to switch between local dialect and English in instances where they believed doing so would enable them to express themselves better. All FGDs were facilitated by trained research assistants and the supervisors with local dialect competencies.

Two separate FGDs were undertaken; each session had eight (8) participants with similar experiences. In total, 16 individuals took part in the FGD and each session included both females and males. Participants in the focus group discussion were encouraged to give their opinion on the questions that will be posed to the group based on their personal experiences and respective community interactions. If they did not wish to answer or were uncomfortable with any of the questions or take part in any of the discussions, they were encouraged to say so. All FGDs took place at the School of Allied Health Science conference room II (SAHS-Room II), University of Cape Coast. The FGD guide was adapted from a previous study [13] to obtain responses to research questions. The key areas of the FGD guide were (a) participants’ description of what anaemia is, (b) individuals at higher risk of developing anaemia, (c) causes of anaemia (d) the consequences of having anaemia, and (d) anaemia prevention strategies (see supplementary data SF2).

All five (5) research assistants (two females & three males) were trained in the ethics guiding the moderation and facilitation of FGDs. Prior to the start of data collection, the FGD guide was piloted among some medical laboratory students, university of Cape Coast. The rationale for the piloting was to provide experiential training in FGDs to the research assistants, assess responses to the questions, and modify the FGD guide based on responses obtained. All FGDs were audio recorded with permission from the participants.

### Data processing/analysis

Quantitative data was analyzed and presented as proportions using Statistical Package for Social Science (SPSS) version 20 for Windows ((IBM SPSS, U.S.A.). In the data analyses presented in Tables 1, 2, 3 and 4 as well as supplementary table S2, the strongly agree and agree categories were merged as agree, whereas strongly disagree and disagree were also merged as “disagree” to ultimately provide three scales i.e., disagree, uncertain, and agree. However, in the structural equation model, the five item Likert scales were maintained. The themes that emerged from the questionnaire data analysis served as the guide for the two (2) focus group discussions. Data obtained from the Focus group discussion was thematically analyzed to identify common themes that have been reported in this study.

**Table 1:** Dietary habits of students

Variable	Total (%)	Gender, n (%)		*p*-value	Age, years; n (%)				*p*-value
		Female	Male		15 – 19	20 – 29	30 – 39	≥ 40	
**I mostly eat out while on campus**				** < 0.001**					**0.027**
Disagree	311 (57.2)	179 (66.8)	132 (47.8)		12 (50.0)	225 (59.8)	63 (52.5)	11 (45.8)	
Uncertain	61 (11.2)	29 (10.8)	32 (11.6)		6 (25.0)	42 (11.2)	11 (9.2)	2 (8.4)	
Agree	171 (31.6)	60 (22.4)	112 (31.7)		6 (25.0)	109 (29.0)	46 (38.4)	11 (45.8)	
**I always prepare my meal**				**0.001**					**0.046**
Disagree	98 (18.0)	36 (13.5)	62 (22.4)		1(4.2)	58 (15.4)	31 (25.8)	8 (33.3)	
Uncertain	46 (8.5)	16 (6.0)	30 (10.9)		4(16.7)	33(8.8)	8(6.7)	1 (4.2)	
Agree	399 (73.5)	215 (80.5)	184 (66.7)		19(79.2)	284(75.7)	81(67.5)	15 (62.5)	
**I always have breakfast, lunch, and supper each day**				0.322					**0.004**
Disagree	204 (37.6)	92(34.4)	112(40.5)		9 (37.5)	149 (39.7)	42 (35.0)	4 (16.7)	
Uncertain	129 (23.8)	68(25.5)	61(22.1)		9 (37.5)	90 (24.0)	29 (24.2)	1 (4.2)	
Agree	210 (38.7)	107 (40.0)	103 (37.3)		6 (25.0)	136 (36.3)	49 (40.8)	19 (79.5)	
**I sometimes skip meals**				0.687					0.534
Disagree	102 (18.8)	51(19.0)	51(18.5)		3 (12.5)	67 (17.8)	27 (22.5)	5 (20.8)	
Uncertain	42 (7.7)	18(6.7)	24(8.7)		1 (4.2)	27 (7.2)	11 (9.2)	3 (12.5)	
Agree	400 (73.5)	199(74.2)	201(72.8)		20 (83.4)	282 (75.0)	82 (68.4)	16 (66.7)	
**I always add vegetables to my diet**				**0.007**					**0.017**
Disagree	162 (29.8)	67 (25.0)	95 (34.4)		6 (25.0)	118 (31.4)	34 (28.3)	5 (20.8)	
Uncertain	146 (26.8)	67 (25.0)	79 (28.6)		12 (50.0)	99 (26.3)	33 (27.5)	3 (12.5)	
Agree	236 (43.4)	134 (50.0)	102 (36.9)		6 (25.0)	159 (42.2)	53 (44.2)	16 (66.7)	

The FGDs were transcribed verbatim by trained personnel with prior experience in transcribing qualitative interviews. Transcriptions were done in accordance with previously suggested best practices in qualitative research interviews [15]. The quality and accuracy of the interview transcription were independently assessed by two members of the research teams who listened to the recorded interviews vis-a-viz the interview transcription. Thematic analyses of the transcripts were independently undertaken by two members of the research team, after an initial generation of a priori list for organizing themes based on the research objectives. The coding and analyses were in accordance with published guidelines involving thematic data analysis [16]. In the results section, major and minor themes that best capture shared ideas are presented for illustration purposes.

Structural equation modeling (SEM) was undertaken using Mplus software version 7 (Muthen & Muthen, U.S.A.) to explore the relationship between knowledge about causes, consequences, and individuals at-risk of anaemia and preventive measures students adopt to curb anaemia. Prior to the structural equation modeling (SEM) of the data, the Little’s MCAR test (Chi-square test: 1167.898, DF: 1065, *p*-value: 0.015) was undertaken to explore missing data pattern. Missing data handling was undertaken using an expectation maximization (EM) algorithm with 25 maximum number of iterations. Subsequently, the adequacy of the questionnaire items and scale correlations were tested using the Kaiser–Meyer–Olkin (KMO) test and Bartlett’s test of Sphericity. KMO of test items was determined to be 0.826 (> 0.7 threshold) and Bartlett’s test of Sphericity was statistically significant (*p* < 0.001). The second section of the questionnaire had five constructs; causes of anaemia, individuals at higher risk of anaemia, dietary habits, consequences of anaemia and anaemia prevention strategies. The reliability of each questionnaire construct was assessed through Cronbach’s Alpha; only constructs with Cronbach’s alpha > 0.7 were included in the SEM. In fitting the SEM, the independent variables were knowledge about causes of anaemia and knowledge about individuals at higher risk of developing anaemia, whereas the dependent variables were knowledge about the consequences of anaemia and anaemia prevention strategies. The latent variable structural model provided a good fit for the data to test the hypothesis that knowledge about causes, consequences and individuals at-risk of anaemia are significantly related to preventive measures students adopt to curb anaemia. Model fit parameters were: Chi-Square Test of Model Fit (1330.232; DF: 399, *p*-value < 0.0001; RMSEA (Root Mean Square Error of Approximation) = 0.066; 90% C.I.: 0.062 – 0.069; Comparative fit index (CFI): 0.744; and Standardized Root Mean Square Residual (SRMR): 0.061.

## Results

### Background characteristics of study participants

Supplementary Table ST1 gives the demographic characteristics of participants of the study. The majority (69.1%) of participants were within the 20 – 29 years age group (age range was 15 – 50 years). Males comprised a slight majority (50.7% vs 49.3% females). Moreover, majority of the participants were single (80.9%), undergraduate students (85.8%) or regular students (70.4%). Overall, only 30.9% of the participants were studying in health and allied sciences programmes.

### Dietary habits of students

The study solicited information on the dietary habits adopted by students while on campus (Table 1). Whereas 73.5% agreed to preparing their meals while on campus, 73.5% indicated skipping meals. Less than half of the students indicated regularly taking three meals a day (38.7%), or regularly including vegetables to their meals (43.4%). When the data was stratified by gender, a significantly higher proportion of females prepared their meals (80.5% vs 66.7%; *p* = 0.001) or included vegetables in their diet (50.0% vs 36.9%; *p* = 0.007) compared to males. Furthermore, a significantly higher proportion of students in the ≥ 40 years age (*p* = 0.027) category indicated regularly taking three square meals or adding vegetables to their diet (*p* = 0.017). A significant proportion of participants in the ≥ 40 years age (*p* = 0.046) category indicated not eating out while they were on campus, but instead prepared their own meals.

### Knowledge on risk of anaemia

Students’ knowledge about individuals at high risk of developing anaemia are shown in Table 2. Overall, 85.5% of the students identified anaemia as a condition resulting from low blood levels. Significantly higher proportions of females agreed that pregnant women (78.0% vs 65.1% males), adolescent females (61.2% vs 47.4% males), and infants (62.2% vs 55.3% males) were at an increased risk of anaemia. In contrast, a significantly higher proportion of males (61.6% vs 52.1% females) agreed that the aged are at an increased risk of anaemia. Moreover, the proportions of females and males that agreed that shorter inter-pregnancy intervals, or vegetarians were risk factors for developing anaemia were comparable. When the data was stratified by age groups, a significantly higher proportion of the students aged ≥ 40 years agreed that pregnant women (91.7%, *p* = 0.001), breastfeeding mothers (75.0%; *p* = 0.001), or women with shorter inter-pregnancy intervals (58.3%; *p* = 0.02) were at increased risk of developing anaemia.

**Table 2:** Students’ knowledge about individuals at risk of anaemia

Variable	Gender, n (%)		*p*-value	Age, years; n (%)				*p*-value
	Female	Male		15 – 19	20 – 29	30 – 39	≥ 40	
**Pregnant women**			**0.004**					**0.001**
Disagree	23 (8.6)	40 (14.6)		1 (4.2)	47 (12.5)	13 (10.8)	2 (8.3)	
Uncertain	36 (13.4)	56 (20.4)		11 (45.8)	69 (18.4)	12 (10.0)	0 (0.0)	
Agree	209 (78.0)	179 (65.1)		12 (50.0)	259 (69.1)	95 (79.2)	22 (91.7)	
**Breastfeeding mothers**			0.156					**0.001**
Disagree	35 (13.1)	52 (18.9)		6 (25.0)	65 (17.3)	13 (10.8)	3 (12.5)	
Uncertain	90 (33.6)	81 (29.5)		11 (45.8)	131 (34.9)	26 (21.7)	3 (12.5)	
Agree	143 (53.4)	142 (51.7)		7 (29.2)	179 (47.7)	81 (67.5)	18 (75.0)	
**Adolescent females**								0.209
Disagree	44(16.4)	68(24.8)	**0.004**	4 (16.7)	83 (22.2)	19 (15.8)	6 (12.5)	
Uncertain	60(22.4)	76(27.7)		6 (25.0)	97 (25.9)	29 (24.2)	4 (16.7)	
Agree	164(61.2)	130(47.4)		14 (58.3)	194 (51.9)	72 (60.0)	14 (70.8)	
**Infants**			** < 0.001**					0.082
Disagree	29 (10.9)	65 (23.6)		5 (20.8)	72 (19.3)	16 (13.3)	1 (4.2)	
Uncertain	72 (27.0)	58 (21.1)		8 (33.3)	95 (25.4)	22 (18.3)	5 (20.8)	
Agree	166 (62.2)	152 (55.3)		11 (45.8)	207 (55.3)	82 (68.3)	18 (75.0)	
*Women with shorter inter-pregnancy intervals								**0.002**
Disagree	34(12.7)	45(16.3)		1 (4.2)	54 (14.4)	18 (15.0)	6 (25.0)	
Uncertain	120 (44.8)	114 (41.5)		20 (83.3)	46 (38.3)	4 (20.0)	4 (16.7)	
Agree	114 (42.5)	116 (42.2)		3 (12.5)	56 (46.7)	11 (55.0)	14 (58.3)	
**Aged people**			**0.045**					0.307
Disagree	34 (12.8)	35 (12.8)		3 (12.5)	45 (12.1)	15 (12.5)	6 (25.0)	
Uncertain	94 (35.2)	70 (25.6)		10 (41.7)	113 (30.3)	36 (30.0)	5 (20.0)	
Agree	139 (52.1)	168 (61.5)		11 (45.8)	215 (57.6)	69 (57.5)	12 (55.0)	
**Vegetarians**			0.422					0.126
Disagree	114 (42.5)	126 (45.8)		13 (54.2)	168 (44.8)	48 (40.0)	11 (45.8)	
Uncertain	91 (34)	79 (28.7)		9 (37.5)	119 (31.7)	38 (31.7)	4 (16.7)	
Agree	63 (23.5)	70 (25.5)		2 (8.3)	88 (23.5)	34 (28.3)	9 (37.5)	

^*^Inter-pregnancy intervals < 18 months was defined as shorter inter-pregnancy intervals

### Knowledge on the consequences of anaemia

Table 3 shows students’ knowledge regarding the possible consequences of anaemia. The majority of the participants agreed that anaemia could impair the mental (61.8%) and physical (72.6) development of children, cause intrauterine death of babies (63.5%), negatively affect academic performance (64.0%), or low birth weight in children (65.4%). However, less than 50% of participants agreed that anaemia could lead to memory loss in adults. When the data was stratified by gender, there were no statistically significant differences in the responses between males and females. Furthermore, stratification of the data by age categories showed that knowledge about the consequences of anaemia was not significantly different except for the impact of anaemia on pregnancy outcomes where 41.7% of teenagers compared to > 60% (*p* = 0.007) in the other age categories indicated anaemia as a potential cause of child loss during pregnancy.

**Table 3:** Students’ knowledge about the consequences of anaemia

Variable	Total, n (%)	Gender, n (%)		*p*-value	Age, years; n (%)				*p*-value
		Female	Male		15 – 19	20 – 29	30 – 39	≥ 40	
**Impairs the mental development of children**				ns					ns
Disagree	69 (12.7)	37 (13.8)	32 (11.6)		2 (8.3)	50 (13.3)	13 (10.8)	4 (16.7)	
Uncertain	139 (25.6)	75 (28.0)	64 (23.2)		6 (25.0)	98 (26.1)	32 (26.7)	3 (12.5)	
Agree	336 (61.8)	156 (58.2)	180 (65.2)		16 (66.7)	228 (60.6)	75 (62.5)	17 (70.8)	
**Impairs the physical development of children**				ns					
Disagree	49 (9.0)	26 (9.7)	23 (8.3)		1 (4.2)	40 (10.7)	7 (5.8)	1 (4.2)	
Uncertain	100 (18.4)	55 (20.6)	45 (16.3)		4 (16.7)	72 (19.2)	22 (18.3)	2 (8.3)	
Agree	394 (72.6)	186 (69.7)	208 (75.4)		19 (79.2)	263 (70.1)	91 (75.8)	21 (87.5)	
**Memory loss in adults**				ns					
Disagree	67 (12.3)	37 (13.8)	30 (10.9)		4 (16.7)	45 (12.0)	17 (14.2)	1 (4.2)	
Uncertain	228 (42.0)	120 (44.8)	108 (39.8)		13 (54.2)	163 (43.5)	47 (39.2)	5 (20.8)	
Agree	248 (45.7)	111 (41.4)	137 (49.8)		7 (29.2)	167 (44.5)	56 (46.7)	18 (75.0)	
**Affects the academic performance of students**				ns					ns
Disagree	56 (10.3)	22 (8.2)	34 (12.4)		2 (8.3)	37 (9.9)	16 (13.4)	1 (4.2)	
Uncertain	139 (25.6)	68 (25.5)	71 (25.8)		6 (25.0)	108 (28.8)	23 (19.3)	2 (8.3)	
Agree	347 (64.0)	177 (66.3)	170 (61.8)		16 (66.7)	230 (61.3)	80 (67.2)	21 (87.5)	
**Loss of child during pregnancy**				ns					**0.007**
Disagree	46 (8.5)	20 (7.5)	26 (9.5)		3 (12.5)	29 (7.7)	12 (10.0)	2 (8.3)	
Uncertain	152 (28.0)	72 (26.9)	80 (29.1)		11 (45.8)	118 (31.5)	19 (15.8)	4 (16.7)	
Agree	345 (63.5)	176 (65.7)	169 (61.5)		10 (41.7)	228 (60.8)	89 (74.2)	18 (75.0)	
**Causes low birth weight in babies**									ns
Disagree	40 (7.4)	18 (6.7)	22 (8.0)		1 (4.2)	27 (7.2)	11 (9.2)	1 (4.2)	
Uncertain	148 (27.2)	77 (28.7)	71 (25.7)		9 (37.5)	114 (30.3)	21 (17.5)	4 (16.7)	
Agree	356 (65.4)	173 (64.6)	183 (66.3)		14 (58.3)	235 (62.5)	88 (73.3)	19 (79.2)	

### Knowledge of students regarding anaemia prevention strategies

The knowledge of students regarding anaemia prevention strategies are presented in Table 4. Majority of the participants agreed that increasing vegetable intake (80.5%), taking iron supplementation (86.4%), or sleeping under mosquito nets (56.5%) are strategies to prevent anaemia. Participants also indicated that frequently deworming (45.9%), preventing teenage pregnancy (30.6%), ensuring ≥ 18 months inter-pregnancy intervals (36.1%) or exclusive breastfeeding for first six months of life (45.1%) could prevent anaemia. When the data was stratified by gender, the knowledge about anaemia prevention strategies did not significantly differ between males and females. However, when the data was stratified by age groups, significantly higher proportion of participants in the ≥ 40 years age group indicated that preventing teenage pregnancy (54.2% vs ≤ 35.0% in the other age categories; *p* = 0.044), or ensuring ≥ 18 months inter-pregnancy intervals (62.5% vs ≤ 45.0% in the other age categories; *p* = 0.001) could prevent anaemia.

**Table 4:** Students’ knowledge about strategies to prevent anaemia

Variable	Total, n (%)	Gender, n (%)		*p*-value	Age, years; n (%)				*p*-value
		Female	Male		15 – 19	20 – 29	30 – 39	≥ 40	
**Increasing vegetable intake at meals time**				ns					ns
Disagree	27 (5.0)	12 (4.5)	15 (5.4)		1 (4.2)	18 (4.8)	7 (5.8)	1 (4.2)	
Uncertain	79 (14.5)	41 (153.2)	38 (13.8)		6 (25.0)	59 (15.7)	10 (8.3)	4 (16.7)	
Agree	438 (80.5)	268 (80.2)	223 (80.8)		17 (70.8)	299 (79.5)	103 (85.8)	19 (79.2)	
**Frequently deworming**				ns					ns
Disagree	104 (19.2)	53 (19.9)	51 (18.5)		6 (25.0)	68 (18.1)	11 (9.2)	1 (4.2)	
Uncertain	190 (35.0)	99 (37.1)	91 (33.0)		11 (45.8)	39 (32.5)	21 (17.5)	4 (16.7)	
Agree	249 (45.9)	115 (43.1)	134 (48.6)		7 (29.2)	56 (46.7)	88 (73.3)	19 (79.2)	
**Taking iron supplementation**				ns					
Disagree	18 (3.3)	9 (3.4)	9 (3.3)		0 (0.0)	12 (3.2)	3 (2.5)	3 (12.5)	
Uncertain	56 (10.3)	29 (10.8)	27 (9.8)		4 (16.7)	41 (10.9)	10 (8.3)	1 (4.2)	
Agree	470 (86.4)	230 (85.8)	240 (87.0)		20 (83.3)	(85.9)	(89.2)	(83.3)	
**Sleeping under mosquito nets**				ns					ns
Disagree	109 (20.1)	52 (19.5)	57 (20.7)		8 (34.8)	71 (18.9)	25 (20.8)	5 (20.8)	
Uncertain	127 (23.4)	62 (23.3)	65 (23.6)		4 (17.4)	97 (25.9)	24 (20.0)	2 (8.3)	
Agree	306 (56.5)	152 (57.1)	154 (55.8)		11 (47.8)	207 (55.2)	71 (59.2)	17 (70.8)	
**Preventing teenage pregnancy**				ns					**0.044**
Disagree	173 (31.9)	91 (34.0)	82 (29.8)		7 (29.2)	121 (32.3)	36 (30.0)	9 (37.5)	
Uncertain	204 (37.6)	84 (31.3)	120 (43.6)		10 (41.7)	150 (40.0)	42 (35.0)	2 (8.3)	
Agree	166 (30.6)	93 (34.7)	73 (26.5)		7 (29.2)	104 (27.7)	42 (35.0)	13 (54.2)	
**Ensuring ≥ 18 months inter-pregnancy intervals**				ns					**0.001**
Disagree	100 (18.4)	51 (19.0)	49 (17.8)		8 (33.3)	65 (17.3)	22 (18.3)	5 (20.8)	
Uncertain	247 (45.5)	111 (41.4)	136 (49.5)		11 (45.8)	188 (50.1)	44 (36.7)	4 (16.7)	
Agree	196 (36.1)	106 (39.6)	90 (32.7)		5 (20.8)	122 (32.5)	54 (45.0)	15 (62.5)	
**Exclusive breastfeeding in the first 6 months of life**				ns					ns
Disagree	91 (16.8)	41 (15.3)	50 (18.2)		3 (12.5)	62 (16.5)	21 (17.5)	5 (20.8)	
Uncertain	207 (38.1)	95 (35.4)	112 (40.7)		11 (45.8)	148 (39.5)	46 (38.3)	2 (8.3)	
Agree	245 (45.1)	132 (49.3)	113 (41.1)		10 (41.7)	165 (44.0)	53 (44.2)	17 (70.8)	

### Effect of knowledge about consequences, individuals’ at-risk of anaemia and preventive measures students adopt to curb anaemia

**Fig. 2:**
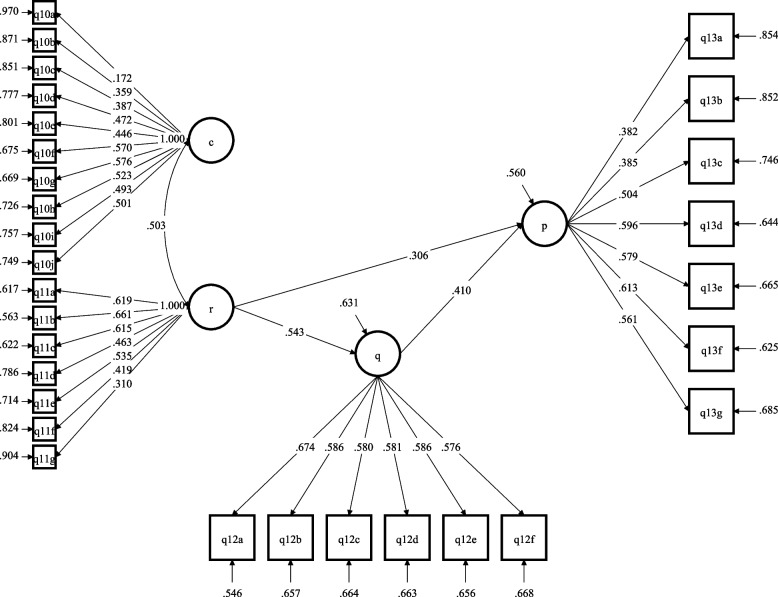
Structural equation model of the effect of knowledge about consequences and individuals’ at-risk groups of anaemia and the preventive measures students adopt to curb anaemia. “c” is the latent variable measuring knowledge about causes of anaemia; “q” is the latent variable measuring knowledge about consequences of anaemia; “r” is latent variable measuring knowledge about individuals at high risk of anaemia; “p” is latent variable measuring knowledge about anaemia prevention strategies. The arrows shown are only significant associations with respective standardized parameter estimates, STDYX. [Model fit parameters were: Chi-Square Test of Model Fit (1330.232; DF: 399, *p*-value = 0.0000; RMSEA (Root Mean Square Error of Approximation) = 0.066; 90% C.I.: 0.062 – 0.069; Comparative fit index (CFI): 0.744; and Standardized Root Mean Square Residual (SRMR): 0.061]

Our latent variable structural model is shown in Fig. 2. Knowing the causes of anaemia did not significantly associate with either knowledge about the consequences of anaemia (b = 0.113, *p* > 0.05) or knowledge about preventive strategies (b = 0.042, *p* > 0.05) that could be adopted to prevent anaemia. However, knowledge about individuals at-risk of anaemia was significantly positively associated with preventive strategies (b = 0.306, *p* < 0.05) that could be employed to curb anaemia. Additionally, knowledge about individuals at-risk of anaemia was significantly positively associated with knowledge about the consequences of anaemia (b = 0.543, *p* < 0.05). Moreover, knowing about the consequences of anaemia seemed to mediate the association between knowledge about at-risk groups and preventive measures that could be adopted to mitigate anaemia since the beta coefficient increased from 0.306 to 0.410 (*p* < 0.05).

### Thematic analysis of FGDs

**Fig. 3:**
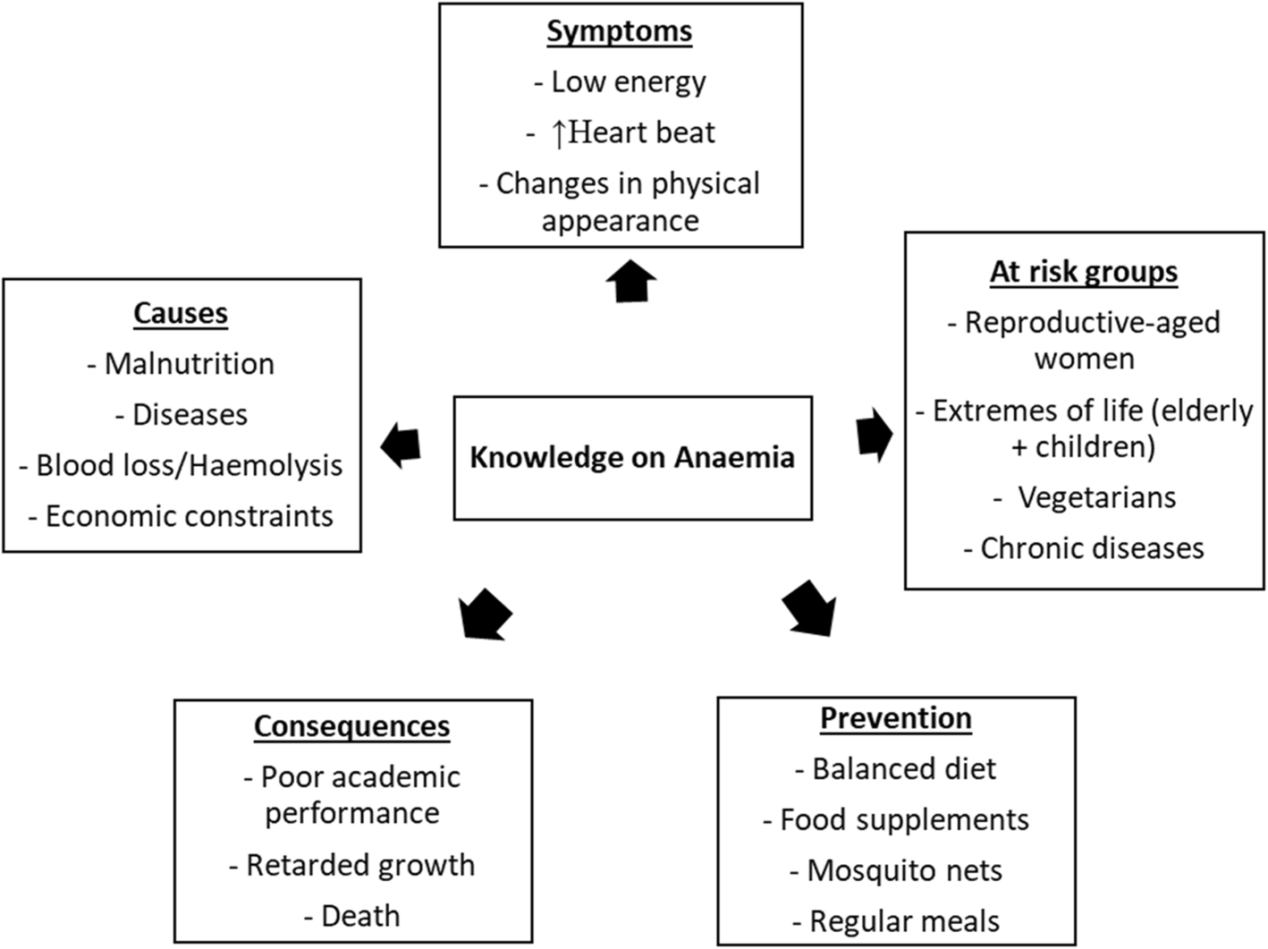
Thematic presentation of FGD on students’ knowledge about anaemia. The priori lists of organizing themes based on research objectives are in bold print; sub-themes that emerged under each theme are listed in the respective box of the thematic area

The FGD was thematically analysed to explore reasons underpinning the choices participants made during the questionnaire item selection (Fig. 3). The major themes that were created from the main constructs are highlighted. The sub-themes that emerged regarding the causes of anaemia included malnutrition, diseases (mainly malaria and sickle cell anaemia), blood loss (bleeding, red cell breakdown, blood loss through childbirth), and economic constraints. Participants also identified poor academic performance, retarded growth, and death as possible consequences of anaemia. Participants opined that regular intake of a balanced diet, taking food supplements as well as sleeping under mosquito net to prevent malaria are important for the prevention of anaemia. Furthermore, participants identified women of reproductive age (major reasons indicated being pregnancy and menstruation), people with chronic diseases, vegetarian lifestyles, and children and the elderly as at-risk groups for anaemia.

## Discussion

Anaemia is estimated to roughly affect one-third of the world’s population and has consistently remained a global health concern [2]. In sub-Saharan Africa, anaemia is a moderate to severe public health problem in most countries [3]. According to a study undertaken by Wiafe and associates in 2021, adolescents in Ghana had less knowledge about iron deficiency anaemia (IDA) or the causes, consequences, and prevention of IDA. Additionally, the Wiafe et al. study also demonstrated that fewer adolescents were knowledgeable about iron-rich food sources or factors that might improve/inhibit iron absorption from diet [17]. Previously, we also showed that anaemia prevalence in a cohort of university students more than doubled in an academic year [10]. The present study therefore employed a mixed-methods approach to model the relationship between tertiary students’ knowledge about individuals at risk of developing anaemia, risks associated with anaemia, causes and consequences of anaemia, and anaemia prevention strategies. Our structural equation modeling demonstrated that students’ knowledge of at-risk groups and the consequences of anaemia significantly influences the preventative strategies that may be adopted to mitigate anaemia. Furthermore, knowledge about the consequences of anaemia significantly positively mediates the impact of knowledge about the at-risk groups on the preventive strategies when considering anaemia from the perspective of our tertiary students’ cohort.

Our study found a disconnect between what the students knew about anaemia and what they did in terms of nutritional choices. Overall, 85.5% of our cohort could correctly identify that anaemia had to do with low levels of blood or haemoglobin concentration. In the focus group discussion, participants were able to identify low energy, easy fatigability, increased heart rate, and changes in physical appearance such as paleness, and yellowish colouration of the eye as potential symptoms of anaemia. These symptoms of anaemia as espoused by our student cohort are consistent with scientific data on symptoms of anaemia [18, 19]. Further, although the majority of the participants knew that increasing vegetable intake could potentially prevent anaemia, this knowledge did not actualize in these students as less than half of the students indicated including vegetables in their diet during their stay on campus. It is instructive to note that during the FGD, the participants suggested economic constraints as a potential cause of anaemia. For example, participants overwhelmingly agreed that the high economic cost of living and poverty makes it difficult to patronize vegetables while on campus. This supports the findings from a multi-national study that showed weak preferences, poverty, and relatively high food prices were important constraints in attaining healthy eating standards in sub-Saharan Africa [20]. In agreement with previous reports, food insecurity precipitated by weak finances, and the financial constraint inherent in higher education make these students prone to unhealthy lifestyle choices. Previous studies have found prevalence of food insecurity to be as high as 44% [21], 39% [22], 38 – 48% [23, 24], respectively among students from University of California, Lebanon, and Australian universities, and therefore suggest a more global problem in the tertiary setting. Furthermore, although our cohort unanimously agreed that regular intake of balanced meals was an important preventive strategy to deal with anaemia, over two-thirds (73.5%) indicated skipping meals while on campus. When the issue of meal skipping was explored during the focus group discussion, the reasons offered for skipping meals were multi-factorial and included demands on time in view of academic work and the high cost of living that make it untenable to routinely eat three-square meals per day. As evidenced in the thematic analysis of the FGD, the students knew that eating a balanced diet that includes meat, egg, vegetables, and fruits, as well as food supplementation was important for anaemia prevention and that failure to routinely take balanced meal would negatively affect academic performance (64.0%). However, the realities on campus are such that they skip meals, and are not able to routinely afford three square meals and thereby predisposing them to anaemia in spite of the knowledge. In support of the link between food insecurity and anaemia predisposition, a synthesis of the literature through a meta-analyses by Moradi and colleagues demonstrated a significant positive association between anaemia or iron deficiency anaemia and food insecurity among various age groups [25]. It is also instructive to note that comparatively, a higher proportion of females (vs males, *p* = 0.007), or participants ≥ 40 years old category (vs lower age groups, *p* = 0.017) indicated regular inclusion of vegetables in their diets. This is suggestive that being female or older was associated with increased healthy eating choices. Although the reason(s) why higher proportion of females saw the importance of vegetables in the diet were not readily apparent, the fact that females generally are responsible for cooking in the traditional Ghanaian family settings, as well as the nutritional education females receive during antenatal clinic visits [26, 27] might be plausible explanations for the observations reported herein.

Noteworthily, about two-thirds of our study cohort could identify potential adverse consequences of anaemia on pregnancy outcomes such as intra-uterine death (63.5%), low birth weight (65.4%) as well as impaired mental (61.8%) and physical (72.6) development of children in agreement with published data [28, 29]. Although this finding is at variance with a previous qualitative study in Ghana where participants were unable to connect anaemia to adverse birth outcomes [13], we believe the differences might stem from the fact that our participants were individuals with tertiary level education (who might have had a higher chance of exposure to anaemia-related literature) whereas < 5% of the Awuah et al., study participants had tertiary level education. In view of these anaemia-induced adverse intra-pregnancy outcomes, as indicated by our study cohort, it was not surprising that more than six-out-of-ten of our study cohort could correctly identify pregnant women, and adolescent females as groups, particularly at high risk of developing anaemia. When participants were asked for clarifications as to why these reproductive aged women were particularly at high risk, FGD participants unanimously opined that the demands of menstruation and pregnancy make these individuals prone to higher anaemia prevalence. These suppositions are in agreement with findings of previous studies that demonstrated that menstruation [30], women of reproductive age [4], and pregnancy [31] are significant determinants of anaemia. Surprisingly however, less than half of our study cohort (42.5%) agreed that shorter inter-pregnancy interval (defined in this study as inter-pregnancy intervals < 18 months) was a significant risk factor for anaemia in reproductive aged women. Even when asked whether ensuring ≥ 18 months inter-pregnancy intervals or preventing teenage pregnancies could be important anaemia prevention strategies in women, only about one-third of the cohort (36.1% and 30.6% respectively) agreed. This contrasts with established scientific data that demonstrate that both teenage pregnancies and shorter inter-pregnancy intervals are each independently associated with adverse maternal and perinatal outcomes [32–34]. Taken together, these findings, therefore demonstrate a lack of comprehensive understanding of the multi-dimensionality of factors that could put an individual at a higher risk of anaemia.

Taken together with previous studies on anaemia [13, 35], our present study is suggestive that poor knowledge or incomplete knowledge about anaemia is an entrenched public health problem that requires a multi-sectorial stakeholder engagement to evolve a holistic public health campaign to increase chances of making any meaningful impact. Bearing in mind that our cohort was young adults who might be in the process of making reproductive choices, it will be interesting to study the lifestyle and dietary choices these young adults consider appropriate in preventing anaemia in the unborn. This would be a worthwhile study considering that less than half of our study cohort agreed that inadequate meat (31.3%) or fish intake (36.2%), road traffic accidents (37.5%) or poor hygiene (40.9%) could potentially cause anaemia (Supplementary Table ST2). If the causative agents of anaemia are poorly understood and/or not appreciated by these tertiary students (some of who were postgraduate students), we postulate a graver anaemia knowledge among individuals with only secondary education or no formal education in the communities. This poor understanding might provide insights as to why anaemia has been consistently a moderate-to-severe public health problem in most sub-Saharan African countries. We propose that a national multi-sectorial stakeholder engagement involving the Ghana health services, the ministry of health, the ministry of education and the national commission for civic education (NCCE) as well as sponsors from the media landscape should be convened to formulate anaemia sensitization strategies, and educational campaigns to tackle the persistent public health threat posed by anaemia. Since the NCCE is the legal body mandated to take policy issues to the grassroots, we consider their involvement to be a priority policy consideration since it would provide an avenue to engage the public through indigenous dialect to help address misconceptions as well offer comprehensive holistic knowledge on anaemia to the populace. It has been noted that the success of policy implementation is greatly influenced by the knowledge of the recipients and the adequacy of collaboration between stakeholders [36]. Additionally, such undertakings should be backed by the needed socioeconomic and macroeconomic empowerment from governments to improve living standards to increase chances of success since social determinants of health are an important consideration in dealing with anaemia [37–39]. In view of our finding of food insecurity among our student cohort, and the reported adverse consequences of food insecurity among tertiary students such as poor mental health [40, 41], and poor academic performance [40], these public health campaigns should consider financial aid and other support systems that could potentially improve food security in tertiary settings.

Our structural equation modeling provided some clarifications about the underlying relationship among the constructs used to explore students’ knowledge about anaemia. Specifically, our study found that knowing the causes of anaemia did not significantly associate with either knowledge about the consequences of anaemia or the knowledge about preventive strategies that could be adopted to prevent anaemia. However, knowledge about individuals at-risk of anaemia was significantly positively associated with preventive strategies that could be employed to curb anaemia. Additionally, knowledge about individuals at-risk of anaemia was significantly positively associated with knowledge about the consequences of anaemia. Importantly, our findings suggest a potential mediating influence of knowledge about the consequences of anaemia on the significant positive association between knowledge about at-risk groups and preventive measures that could be adopted to mitigate anaemia. The fact that simply knowing about anaemia did not significantly associate with the other constructs suggests a possible complex, non-linearity of relationships between the constructs. Taken together, we propose that any public health campaign seeking to address anaemia in sub-Saharan should adopt the complex adaptive systems (CAS) thinking approach [42, 43] given the plausible inter-relating personal, political and socio-economic factors that may cumulatively impact policy outcomes. Such a CAS approach would be invaluable in unearthing potential feedback loops (positive and/or negative), as well as factors that may cross multiple domains and stimulate consensus building among stakeholders to ensure success with policy implementation.

Although this study employed qualitative and quantitative data collection processes to increase the rigour of the study outcomes, we acknowledge some limitations. Our sampling frame covered only tertiary students which might have introduced an element of selection bias in view of the fact that educational attainment is an important consideration in the social determinants of disease conditions [39, 44]. The present study employed convenience sampling technique in which only students who agreed to participate were recruited; this might have likely introduced participation bias in the data presented herein. Additionally, our study is a one-centre study that involved only students from the University of Cape Coast and may not necessarily be generalizable to other tertiary institutions across the country since the dynamics differs across the country. Furthermore, in view of the exclusion of students on the distance learning mode from the study, we acknowledge that the findings may not reflect the experiences and anaemia knowledge among such students considering that food insecurity issues may not be as pronounced among such students. In spite of these limitations, we are of the opinion that since the University of Cape Coast is an equal access university and admits students from all across the 16 regions of Ghana, and the fact that the sampling cut across colleges offering health and non-health related programmes, our data provides an approximation of the anaemia knowledge base among young adults in Ghana.

### Supplementary Information

**Additional file 1: Supplementary File SF1.** Anaemia knowledge study questionnaire.** Supplementary File SF2.** Focus group discussion guide for study on university of cape coast students’ knowledge about anaemia.** Supplementary Table ST1.** Demographic characteristics of participants.** Supplementary Table ST2.** Students’ knowledge about causes of anaemia.

## Data Availability

The datasets used and/or analysed during the current study are available from the corresponding author on reasonable request.

## Abbreviations

FGD: Focus group discussion
SEM: Structural equation modeling
IDA: Iron deficiency anaemia
CAS: Complex adaptive systems
