# Supplementary material for: Exploring the perspective of young adults about anaemia prevention; the contributions of knowledge about at-risk groups and consequences of anaemia
Source: BMC Public Health. 2023 Oct 24;23:2081. doi: 10.1186/s12889-023-16980-2 (PMC10598987; doi:10.1186/s12889-023-16980-2)
Supplement: Supplementary file 1 — Additional file 1: Supplementary File SF1. Anaemia knowledge study questionnaire. Supplementary File SF2. Focus group discussion guide for study on university of cape coast students’ knowledge about anaemia. Supplementary Table ST1. Demographic characteristics of participants. Supplementary Table ST2. Students’ knowledge about causes of anaemia. [file 12889_2023_16980_MOESM1_ESM.docx]

***Supplementary File SF1: Anaemia knowledge study Questionnaire***

**COVER LETTER**

We are interested in learning about your understanding regarding a condition called **anaemia among university student**. Anaemia has persistently been one of the commonest conditions in Ghana, suggesting that perhaps public education is very low. The study is in two phases: Questionnaire survey and focus group discussion (this section is for only those who want to join in the discussion on **anaemia**). The findings of the study shall be useful in understanding the knowledge level among university students, causes and prevention of the youth regarding **anaemia**. Therefore, the findings may be published in international journal to increase access to the findings.

### As the study is voluntary, your participation very much appreciated. Your responses shall be kept anonymous and confidential. Since there are no right or wrong answers, please do your best to fill the questionnaire as honestly as possible to reflect your opinion and experience. Please indicate on the questionnaire if you would want to be part of the focus group discussion where we will share ideas about anaemia with other students. The questionnaire will take between 5 – 10 minutes of your time.

Thank you for participating in the study.

**PART I: BACKGROUND DATA**

**Path number: __________________________**

1. Age (years): _______________ years
2. Sex: [ ] Female [ ] Male
3. Marital status: [ ] Married [ ] Single [ ] Divorced [ ] Widowed [ ] Cohabiting
4. Highest educational attainment: [ ] Primary [ ] Secondary [ ] Vocational [ ] Undergraduate
5. Vocation: [ ] Schooling [ ] Learning a trade [ ] Others, ___________
6. **Email address/phone number**: *(this is optional) * for those who are willing to be part of focus group discussion:* _______________________________________

**PART 2: KNOWLEDGE ABOUT CAUSES OF ANAEMIA**

1. Which of the following best describes your understanding of anaemia?

[ ] When a person has low blood levels

[ ] When a person has low energy

[ ] When a person has high blood cholesterol level

[ ] Other, please specify ______________________________________________

1. How likely do you think you are to be **anaemic**?

[ ] Not likely

[ ] You are not sure

[ ] Likely

1. How much does each of the following describes your **dietary habits/practices** on campus

|  | Strongly disagree (1) | Disagree (2) | Uncertain (3) | Agree (4) | Strongly agree (5) |
| --- | --- | --- | --- | --- | --- |
| I mostly eat out while on campus |  |  |  |  |  |
| I always prepare my own meals |  |  |  |  |  |
| I always have breakfast, lunch and supper each day |  |  |  |  |  |
| I sometimes skip meals |  |  |  |  |  |
| I always add vegetables to my diet |  |  |  |  |  |

1. Please tick below to show how much you agree or disagree with each of the following as a **cause of anaemia**

|  | Strongly disagree (1) | Disagree (2) | Uncertain (3) | Agree (4) | Strongly agree (5) |
| --- | --- | --- | --- | --- | --- |
| Being a vegetarian |  |  |  |  |  |
| Inadequate fish intake |  |  |  |  |  |
| Inadequate meat intake |  |  |  |  |  |
| Infections/sickness |  |  |  |  |  |
| Road traffic accidents |  |  |  |  |  |
| Inadequate vegetable intake |  |  |  |  |  |
| Poverty |  |  |  |  |  |
| Low education attainment |  |  |  |  |  |
| Poor hygiene |  |  |  |  |  |
| Inherited genetic diseases |  |  |  |  |  |

1. How much do you agree or disagree concerning which of these groups are **at risk of developing anaemia**?

|  | Strongly disagree (1) | Disagree (2) | Uncertain (3) | Agree (4) | Strongly agree (5) |
| --- | --- | --- | --- | --- | --- |
| Pregnant women |  |  |  |  |  |
| Breastfeeding mothers |  |  |  |  |  |
| Adolescent females |  |  |  |  |  |
| Infants |  |  |  |  |  |
| Women with short inter-pregnancy intervals (<18 months, that is 1 year and six months) |  |  |  |  |  |
| Aged people |  |  |  |  |  |
| Vegetarians |  |  |  |  |  |

**PART 3: CONSEQUENCES OF ANAEMIA**

|  | Strongly disagree (1) | Disagree (2) | Uncertain (3) | Agree (4) | Strongly agree (5) |
| --- | --- | --- | --- | --- | --- |
| Anaemia impairs mental development in children |  |  |  |  |  |
| Anaemia impairs physical development in children |  |  |  |  |  |
| Anaemia may lead to memory loss in adults |  |  |  |  |  |
| Anaemia affects academic performance |  |  |  |  |  |
| Anaemia may lead to loss of child in pregnancy |  |  |  |  |  |
| Anaemia causes low birth weight in babies |  |  |  |  |  |

**PART 4: PREVENTION OF ANAEMIA**

Please indicate by ticking (**√**) how much you agree or disagree with the following regarding anaemia prevention.

|  | Strongly disagree (1) | Disagree (2) | Uncertain (3) | Agree (4) | Strongly agree (5) |
| --- | --- | --- | --- | --- | --- |
| Frequently deworming |  |  |  |  |  |
| Taking iron supplementation |  |  |  |  |  |
| Increasing vegetable intake at meal times |  |  |  |  |  |
| Sleeping under mosquito treated nets |  |  |  |  |  |
| Preventing teenage pregnancy |  |  |  |  |  |
| Ensuring that the time between successive pregnancies is at least 18 months (1 year and six months) |  |  |  |  |  |
| Exclusive breastfeeding in the first 6 months of a child’s life. |  |  |  |  |  |

***Supplementary File SF2:*** ***Focus group discussion guide for study on university of cape coast students’ knowledge about anaemia.***

Thank you for honouring our invitation to join this focus group discussion. Today, we are going to be talking about anaemia. Anaemia has persistently been one of the commonest conditions in Ghana, suggesting that perhaps public education is very low. So, our focus is to explore the UCC students’ knowledge and understanding of what anaemia is, the causes of anaemia, consequences, as well as look at its effects and preventive measures. We are going to be tape-recording this discussion for the purposes of transcribing the discussion and to assist in the subsequent data analyses. There are no right or wrong answers; therefore, we encourage everyone to freely contribute. We expect our discussion to last about 45 – 60 minutes. We expect our discussion to be guided by these questions.

1. Can you describe what anaemia is?

**Probe**: How is anaemia understood in the community?

1. Who is most likely to get anaemia?

**Probe:** Young children, adolescent girls, women, men, etc.?

1. What do you think causes anaemia?

**Probe:** why do participants consider a particular group to be at risk?

1. What are the consequences of having anaemia?
2. What can be done to prevent anaemia?

***Supplementary Table ST1: Demographic Characteristics of Participants***

| **Characteristic** |  | N (%) |
| --- | --- | --- |
| **Age (years)** | 15 – 19 | 24 (4.4) |
|  | 20 – 29 | 376 (69.1) |
|  | 30 – 39 | 120 (22.1) |
|  | ≥40 | 24 (4.4) |
| **Gender** | Female | 268 (49.3) |
|  | Male | 276 (50.7) |
| **Marital status** | Married | 54 (9.9) |
|  | Single | 440 (80.9) |
|  | Divorced | 45 (8.3) |
|  | Widowed | 2 (0.4) |
|  | Cohabiting | 1 (0.2) |
| **Educational level** | Undergraduate | 467 (85.8) |
|  | Masters | 73 (13.4) |
|  | Doctoral | 3 (0.6) |
| **Student type** | Regular | 383 (70.4) |
|  | Sandwich | 161 (29.6) |
| **Which college do you belong to?** | Humanities and Legal studies | 127 (33.2) |
|  | Education studies | 60 (15.7) |
|  | Agricultural and natural sciences | 77 (20.2) |
|  | Health and allied sciences | 118 (30.9) |

***Supplementary Table ST2: Students’ knowledge about causes of anaemia***

| **Variable** | **Total, n (%)** | **Gender, n (%)** | | **p-value** | **Age, years; n (%)** | | | | **p-value** |
| --- | --- | --- | --- | --- | --- | --- | --- | --- | --- |
|  |  | Female | Male |  | 15 – 19 | 20 – 29 | 30 – 39 | ≥40 |  |
| **Being vegetarian** | | | | ns |  |  |  |  |  |
| Disagree | 365 (67.2) | 184 (68.9) | 181 (65.6) |  | 17 (70.8) | 252 (67.0) | 82 (68.9) | 14 (58.3) |  |
| Uncertain | 98 (18.0) | 44 (16.5) | 54 (19.6) |  | 5 (20.8) | 72 (19.1) | 18 (15.1) | 3 (12.5) |  |
| Agree | 80 (14.7) | 39 (14.6) | 41 (14.9) |  | 2 (8.3) | 52 (13.8) | 19 (16.0) | 7 (29.2) |  |
| **Inadequate fish intake** | | | | ns |  |  |  |  | ns |
| Disagree | 233 (42.9) | 115 (43.1) | 118 (42.9) |  | 12 (50.0) | 159 (42.3) | 51 (42.9) | 11 (45.8) |  |
| Uncertain | 140 (25.8) | 71 (26.6) | 69 (25.0) |  | 7 (29.2) | 105 (27.9) | 25 (21.0) | 3 (12.5) |  |
| Agree | 170 (31.3) | 81 (30.3) | 89 (32.2) |  | 5 (20.8) | 112 (29.8) | 43 (36.1) | 10 (41.7) |  |
| **Inadequate meat intake** | | | | ns |  |  |  |  | 0.05 |
| Disagree | 215 (39.7) | 105 (39.5) | 110 (39.9) |  | 10 (43.5) | 151 (40.3) | 43 (35.8) | 11 (45.8) |  |
| Uncertain | 131 (24.2) | 68 (25.6) | 63 (22.8) |  | 9 (39.1) | 97 (25.9) | 21 (17.5) | 4 (16.7) |  |
| Agree | 196 (36.2) | 93 (35.0) | 103 (37.3) |  | 4 (17.4) | 127 (33.9) | 56 (46.7) | 9 (37.5) |  |
| **Infections** | | | | ns |  |  |  |  | ns |
| Disagree | 126 (23.4) | 57 (21.5) | 69 (25.3) |  | 3 (13.0) | 95 (25.5) | 23 (19.3) | 5 (20.8) |  |
| Uncertain | 99 (18.4) | 49 (18.5) | 50 (18.3) |  | 6 (26.1) | 63 (16.9) | 24 (20.2) | 6 (25.0) |  |
| Agree | 313 (58.2) | 159 (60.0) | 154 (56.4) |  | 14 (60.9) | 214 (57.5) | 72 (60.5) | 13 (54.2) |  |
| **Road traffic accident** | | | | ns |  |  |  |  |  |
| Disagree | 239 (44.2) | 122 (45.7) | 117 (42.7) |  | 16 (66.7) | 169 (45.3) | 45 (37.5) | 9 (37.5) | ns |
| Uncertain | 99 (18.3) | 48 (18.0) | 51 (18.6) |  | 5 (20.8) | 68 (18.2) | 21 (17.5) | 5 (20.8) |  |
| Agree | 203 (37.5) | 97 (36.3) | 106 (38.7) |  | 3 (12.5) | 136 (36.5) | 54 (45.0) | 10 (41.7) |  |
| **Poverty** | | | | ns |  |  |  |  |  |
| Disagree | 200 (36.9) | 90 (33.6) | 110 (40.1) |  | 9 (37.5) | 143 (38.2) | 39 (32.5) | 9 (37.5) |  |
| Uncertain | 97 (17.9) | 48 (17.9) | 49 (17.9) |  | 3 (12.5) | 73 (19.5) | 20 (16.7) | 1 (4.2) |  |
| Agree | 245 (45.2) | 130 (48.5) | 115 (42.0) |  | 12 (50.0) | 158 (42.2) | 61 (50.8) | 14 (58.3) |  |
| **Low educational attainment** | | | | ns |  |  |  |  | ns |
| Disagree | 297 (54.6) | 136 (50.7) | 161 (58.3) |  | 11 (45.6) | 213 (56.6) | 62 (51.7) | 11 (45.8) |  |
| Uncertain | 95 (17.5) | 49 (18.3) | 46 (16.7) |  | 7 (29.2) | 66 (17.6) | 18 (15.0) | 4 (16.7) |  |
| Agree | 152 (27.9) | 83 (31.0) | 69 (25.0) |  | 6 (25.0) | 97 (25.8) | 40 (33.3) | 9 (37.5) |  |
| **Poor hygiene** | | | | ns |  |  |  |  | ns |
| Disagree | 184 (34.2) | 92 (35.1) | 92 (33.3) |  | 5 (21.7) | 133 (35.8) | 37 (31.1) | 9 (37.5) |  |
| Uncertain | 134 (24.9) | 63 (24.0) | 71 (25.7) |  | 7 (30.4) | 98 (26.3) | 22 (18.5) | 7 (29.2) |  |
| Agree | 220 (40.9) | 107 (40.8) | 113 (40.9) |  | 11 (47.8) | 141 (37.9) | 60 (50.4) | 8 (33.3) |  |
| **Inherited genetic disease conditions** | | | | ns |  |  |  |  | ns |
| Disagree | 106 (19.5) | 48 (17.9) | 58 (21.0) |  | 3 (12.5) | 71 (18.9) | 24 (20.0) | 8 (33.3) |  |
| Uncertain | 103 (18.9) | 55 (20.5) | 48 (17.4) |  | 8 (33.3) | 79 (21.0) | 14 (11.7) | 2 (8.3) |  |
| Agree | 335 (61.6) | 165 (61.6) | 170 (61.6) |  | 13 (54.2) | 226 (60.1) | 82 (68.3) | 14 (58.3) |  |
